# Case Report: Azathioprine: An Old and Wronged Immunosuppressant

**DOI:** 10.3389/fimmu.2022.903012

**Published:** 2022-06-10

**Authors:** Pedro R. Chocair, Precil Diego Miranda de Menezes Neves, Sara Mohrbacher, Maurilio Pacheco Neto, Victor A. H. Sato, Érico S. Oliveira, Leonardo V. Barbosa, Alessandra M. Bales, Fagner Pereira da Silva, Américo L. Cuvello-Neto, John A. Duley

**Affiliations:** ^1^ Internal Medicine and Nephrology Service, Hospital Alemão Oswaldo Cruz, Sao Paulo, Brazil; ^2^ School of Medicine, Sapucai Valley University, Pouso Alegre, Brazil; ^3^ Nursing Service, Hospital Alemão Oswaldo Cruz, Sao Paulo, Brazil; ^4^ School of Pharmacy, The University of Queensland, Woolloongabba, QLD, Australia

**Keywords:** 6-TGN, azathioprine, mycophenolate, renal transplant, metabolites, allopurinol

## Abstract

Mycophenolate rapidly substituted azathioprine (AZA) in transplant immunosuppression regimens since the 1990s, when early clinical trials indicated better outcomes, although opposite results were also observed. However, none of these trials used the well-established optimization methods for AZA dosing, namely, thiopurine methyltransferase pharmacogenetics combined with monitoring of the thiopurine metabolites 6-thioguanine nucleotides (6-TGN) and 6-methylmercaptopurine (6-MMP). Resistance to optimize AZA therapy remains today in transplant therapy, despite the fact that thiopurine metabolite testing is being used by other medical disciplines with evident improvement in clinical results. In a previous analysis, we found that active 6-TGN metabolites were not detectable in about 30% of kidney transplant patients under continuous use of apparently adequate azathioprine dosage, which demonstrates the need to monitor these metabolites for therapeutic optimization. Two of four case studies presented here exemplifies this fact. On the other hand, some patients have toxic 6-TGN levels with a theoretically appropriate dose, as seen in the other two case studies in this presentation, constituting one more important reason to monitor the AZA dose administered by its metabolites. This analysis is not intended to prove the superiority of one immunosuppressant over another, but to draw attention to a fact: there are thousands of patients around the world receiving an inadequate dose of azathioprine and, therefore, with inappropriate immunosuppression. This report is also intended to draw attention, to clinicians using thiopurines, that allopurinol co-therapy with AZA is a useful therapeutic pathway for those patients who do not adequately form active thioguanine metabolites.

## Introduction

The lymphoid specificity of the thiopurine group of drugs, invented in the early 1960s by Gertrude Elion and George Hitchings (1), was exemplified by the antimetabolite 6-mercaptopurine, which had proven to be successful in treating acute lymphoblastic leukemia. Modification of 6-mercaptopurine led to the development of azathioprine (AZA), the first successful immunosuppressant that opened the era of organ transplantation.

For renal transplantation, AZA was initially used in combination with prednisolone (Pred) as ‘double therapy’, but with the advent of cyclosporine-A (CyA) in the 1970s, a ‘triple therapy’ regimen of AZA/Pred/CyA became standard for two decades. Then in 1995 the antimetabolite mycophenolic acid mofetil (MMF) was approved for clinical as an immunosuppressant and became the most frequently used immunosuppressant for solid organ transplantation, particularly kidney transplantation (2), despite the higher price and significant gastrointestinal complications, which may lead to dose reductions or drug withdrawal.

Another formulation of mycophenolic acid, enteric-coated sodium mycophenolate, was approved for transplantation in 2004 and is currently the immunosuppressant preferred by the vast majority of kidney transplant centers.

Mycophenolate has replaced AZA in immunosuppression regimens, but is it better?

In retrospect, there have been serious deficiencies in virtually all clinical trials comparing the efficacy of AZA with mycophenylate. The absence of initial assessment of the genetic status of thiopurine methyltransferase (TPMT), or therapeutic monitoring of the major thiopurine metabolites, 6-thioguanine nucleotide (6-TGN) and 6-methylmercaptopurine (6-MMP), have been striking oversights. We published the first study using TPMT for renal transplantation in 1992 (3), in patients receiving AZA triple therapy, when we suggested that the wide range of TPMT activity (3, 4) may be an important factor in determining long-term graft survival in AZA-treated patients. At that time, we recommended that those patients with high TPMT activity might benefit from AZA doses near the upper limit. Bergan et al. (5) showed that high-dose AZA therapy following renal transplantation, monitored by 6-TGN, kept myelotoxicity within acceptable limits with the benefit of a reduction in acute rejection episodes.

We first demonstrated the beneficial effect of low dose allopurinol co-therapy with AZA for organ transplantation in 1993 (6). Only one episode of acute rejection was observed among the 12 allopurinol-treated patients, whereas 11 of 12 in a control group had rejection episodes, seven with two or more crises. Other studies have confirmed that prior TPMT assessment, combined in some cases with thiopurine metabolite monitoring, improves the efficacy of AZA for organ transplantation and for other clinical uses.

Previous studies of inflammatory bowel disease (IBD) patients showed that the therapeutic and safe levels of 6-TGN and 6-MMP were 235 - 450 pmol/8x10^8^ red blood cells (RBC), and < 5700 pmol/8x10^8^ RBC respectively, and those reference ranges have been extrapolated to other populations such as renal transplant patients (7, 8). More recently, we have measured 6-TGN in 88 kidney transplant patients, 54 male/34 female, age 27 to 81 years (median 49 years), post-transplant time from 0.4 to 38 years (median 11 years), with serum creatinine variable from 0.6 to 10 mg/dL (median 1.2 mg/dL). Very low or undetectable levels of 6-TGN were observed in approximately 30% of these 88 patients, who were receiving apparently adequate doses of AZA (unpublished data). Those patients were probably “shunters”, however the measurement of 6-MMP and TPMT were not performed. AZA co-therapy with allopurinol, rather than AZA dose escalation, has now become indicated for these patients, who are usually identifiable by their high 6-MMP levels as ‘thiopurine shunters.’ In these patients, AZA is ‘shunted’*via* TPMT into inactive 6-MMP rather than forming active 6-TGN. Allopurinol counteracts this effect (9).

A survey carried out by Fargher et al. (10) showed that by 2007, two-thirds of dermatologists, gastroenterologists and rheumatologists in England were using TPMT genotyping or enzyme-phenotyping prior to AZA treatment. However, therapeutic monitoring of AZA metabolites (6-TGN and 6-MMP) continues to be very rarely used prospectively by clinicians in the management of immunosuppressed patients.

Herein, we report our experience with four kidney transplant patients that highlights the pivotal importance of adjusting the AZA dose by measuring thiopurine metabolites.

## Case Reports

### Case 1 - Recurrent Acute Pancreatitis in a Patient With High 6-TGN Despite Apparently Adequate Dose of AZA

**Figure 1:**
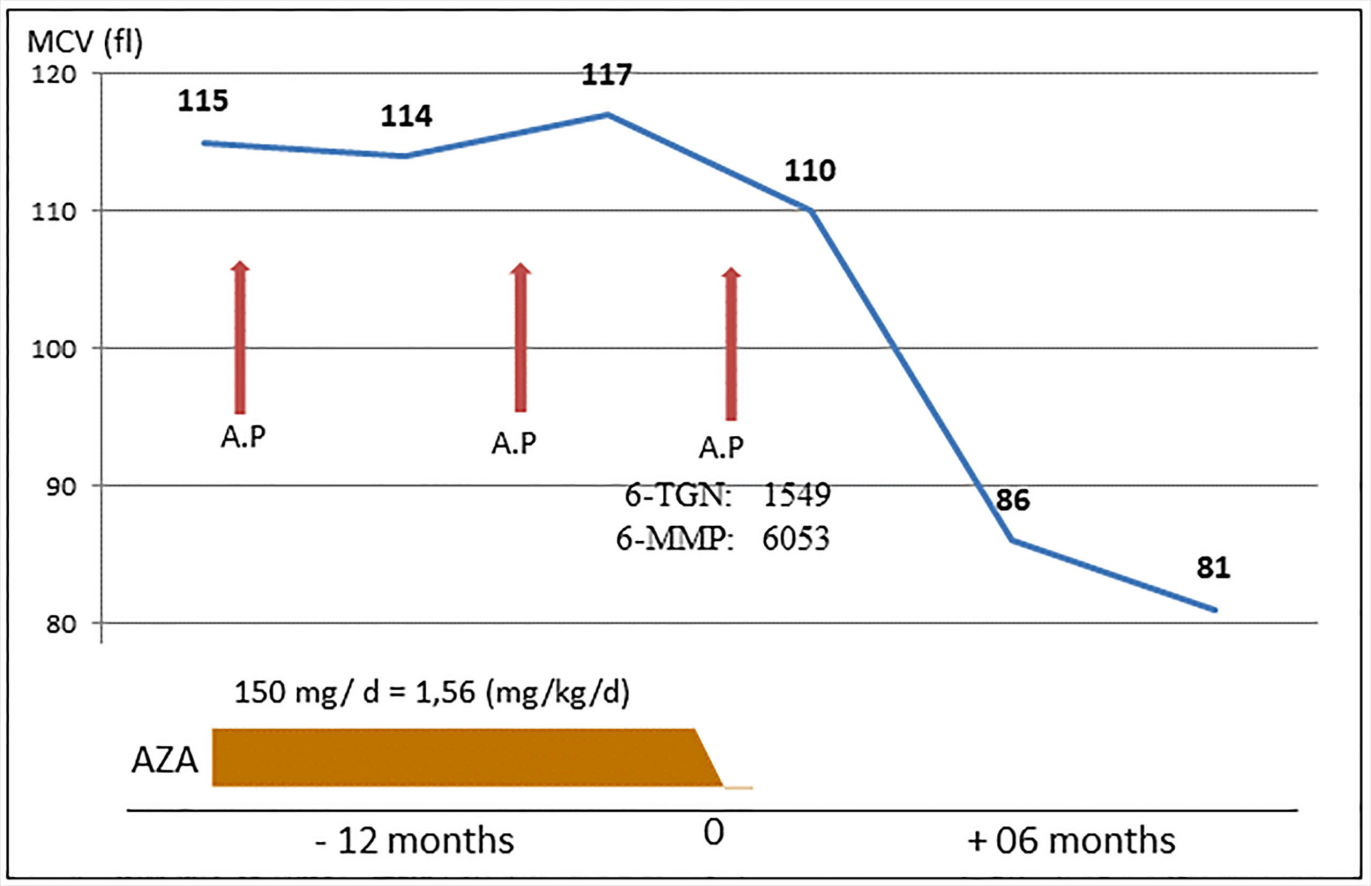
Case 1: Three episodes of pancreatitis related to AZA intake, during an 18-month period. AP, acute pancreatitis.

The **Figure 1** illustrates the clinical history of a 57-year Brazilian male renal transplant patient who remained stable with normal renal function for 30 years on AZA (dosage 150 mg/day) and prednisone (5 mg/day). He presented with three episodes of mild acute pancreatitis. No causal factor was found despite intensive investigation, but AZA was not initially suspected as the causal agent. The suspicion of azathioprine as a causal factor only occurred after the third episode, with the finding of a grossly raised RBC concentration of 6-TGN (1549 pmol/8x10^8^ RBC), as well as mildly raised methylated metabolite 6-MMP (6053 pmol/8x10^8^ RBC), performed by assay with Liquid Chromatography with tandem mass spectrometry (LC-MS-MS), despite the clinically acceptable dose of 1.6 mg/kg. His maintenance immunosuppression regimen was subsequently changed to mycophenolate and tacrolimus.

### Case 2 - Kidney Transplant Patient With Severe AZA-Related Diarrhea Despite Apparently Adequate Dose

**Figure 2:**
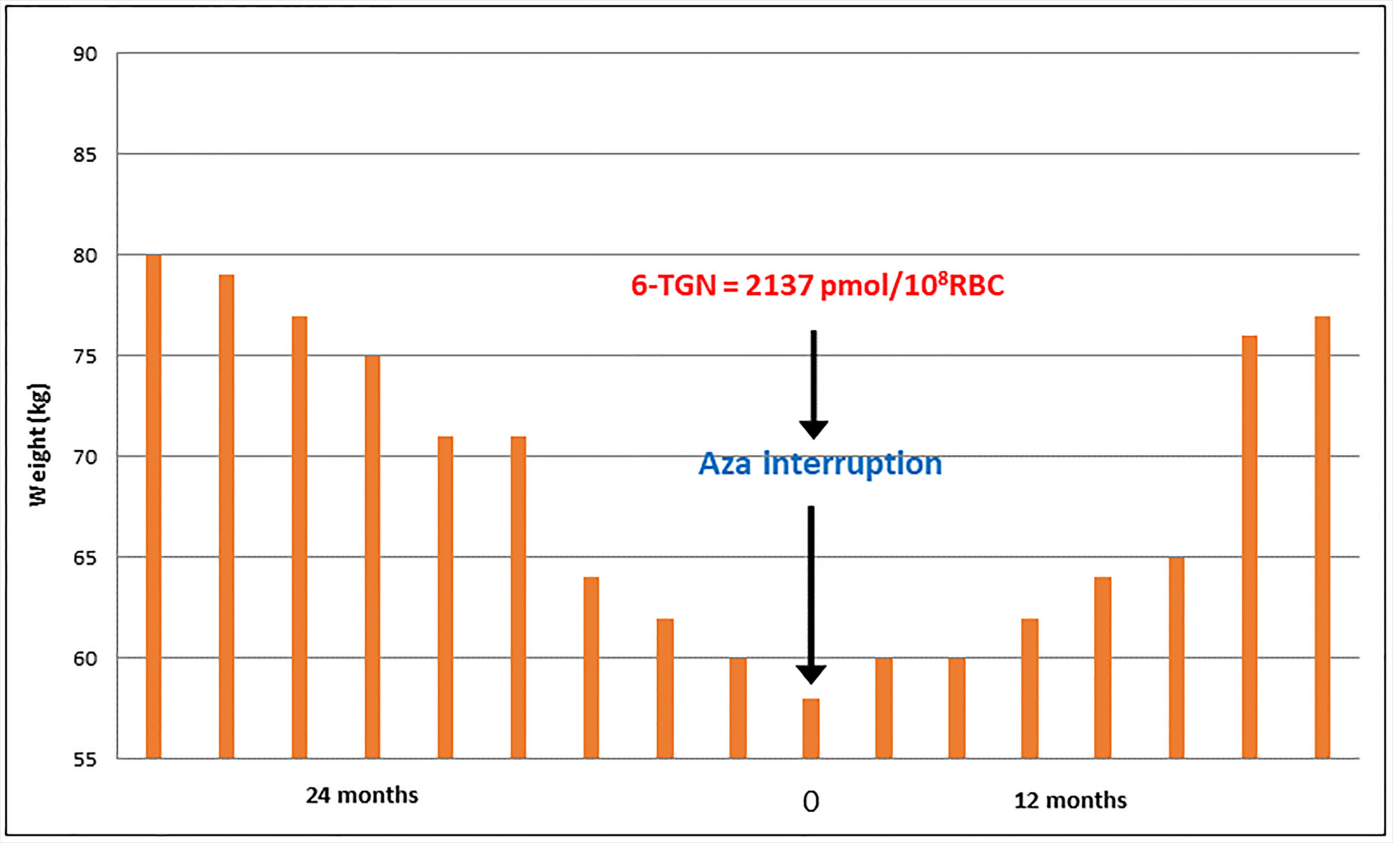
Case 2: Follow-up of patient body weight over a 36-month period, including cessation of AZA intake.

The **Figure 2** shows the results for a 28-year-old male Brazilian renal transplant patient on AZA plus, prednisone with normal renal function (creatinine 1.2 mg/dL), who presented with persistent diarrhea for two years, accompanied by a significant reduction in body weight. Despite extensive investigation, the cause of diarrhea was not found. His AZA dosage at the time of discontinuation was 125mg/day (~2 mg/kg), with very high levels of 6-TGN (2137 pmol/8x10^8^ RBC). The patient’s maintenance immunosuppressive regimen was then changed to tacrolimus and prednisone, and there was a gradual normalization of bowel habit and weight recovery.

### Case 3 - Patient With Undetectable 6 TGN Despite Apparently Adequate Dose of Azathioprine

A 47-year-old kidney transplant patient (cadaver donor) presented with two episodes of histologically proven acute cellular rejection in the first three months post-transplant, despite thymoglobulin induction and AZA/prednisone immunosuppression, plus tacrolimus maintenance (**Table 1**). The red cell 6-TGN levels were measured for the first time during the fourth month post-transplantation, while the patient was receiving 200 mg/day (2.6 mg/kg) azathioprine and prednisone, plus tacrolimus. The 6-TGN was undetectable (<50 pmol/8x10^8^ RBC), so the dosage was repeated, and the previous 6-TGN result was confirmed and dosage of 6-MMP was 1062 pmol/8x10^8^ RBC. To rectify the absence of 6-TGN 10 mg/day allopurinol was added to the therapy, while continuing 200 mg/day AZA. Two and three months later, TGN levels were 203 and 247 pmol/8x10^8^ RBC, respectively. Red cell MCV increased but the white blood cell count remained within an acceptable level, and serum creatinine improved. Current immunosuppression includes tacrolimus, prednisone, AZA (200 mg/day) and allopurinol (10 mg/day), with serum creatinine of 1.5 mg/dL, and the patient remains well.

**Table 1:** Patient with undetectable 6-TGN despite apparently adequate dose of AZA.

Month post-Tx	Creatinine (mg/dL)	WBC (mm^-3^)	MCV (fL)	6-TGN (pmol/8x10^8^)	6-MMP (pmol/8x10)	AZA (mg/d)	Allopurinol (mg/d)
0	5.0	8,300	86.5	–	–	200	0
1	2.4	17,300	89.7	–	–	200	0
2	2.1	13,070	92.3	–	–	200	0
3	2.1	14,680	94.8	< 50	717	200	0
4	1.9	10,820	98.3	< 50	1062	200	10
5	2.0	7,370	102	115	3678	200	10
6	1.8	6,900	100	203	2210	200	10
7	1.6	6,380	105	247	1759	200	10
8	1.5	5,940	108	-	-	200	10
9	–	–	-	-	-	200	10
10	1.5	5,300	107	-	-	200	10
11	1.4	6,670	106	288	3660	200	10

Tx, transplantation; WBC, white blood cell count; MCV, red cell mean corpuscular volume; 6-TGN, red cell thioguanine nucleotide concentration; AZA, azathioprine dosage per day; -, not measured; 6-TGN < 50, below limit of detection.

### Case 4 - Patient With Low 6-TGN Despite Apparently Adequate Dose of AZA

The fourth case was a 28-year-old obese patient (100 kg) with histologically confirmed IgA nephropathy, who underwent preemptive renal transplantation in 2008 with a haplo-identical sister’s kidney. His blood pressure was well controlled with losartan and furosemide. Post-transplant immunosuppression included prednisone, 200 mg/day (2 mg/kg) AZA, and serum-adjusted tacrolimus. The initial dose of prednisone was 50 mg/day, then progressively reduced to a maintenance dose of 5 mg/day from the third month after transplantation.

There was no rejection crisis and creatinine remained stable around 1.4 mg/dL. The patient has gained weight during the post-transplant period, currently weighing 120 kg. He has required antihypertensive therapy (losartan, amlodipine, chlorthalidone), and maintains a satisfactory blood pressure of around 120/80 mm/Hg. During 2021, the patient presented with severe Covid-19, remaining intubated for two weeks before recovering fully. In August 2019, 11 years post-transplant, the measurement of AZA metabolites was included among the routine laboratory tests. **Table 2** summarizes the patient’s immunosuppression evolution during Aug. 2019 to Jan. 2022. An increase in the levels of 6-TGN and MCV was observed after the introduction of 5 mg/day allopurinol. As 6-TGN levels reached levels above normal limits (570 pmol/8x10^8^), the AZA dose was reduced from 200 to 100 mg/day with consequent normalization of the metabolite (293 pmol/8x10^8^).

**Table 2:** Patient with low 6-TGN despite apparently adequate dose of AZA.

Date	Creatinine(mg/dL)	WBC (mm^-3^)	MCV(fL)	6-TGN (pmol/8x10^8^)	6-MMP (pmol/8x10)	AZA (mg/d)	Allopurinol (mg/d)
Aug. 2019	1.3	9,600	92.2	–	–	200	0
Aug 2020	1.4	10,700	90	–	–	200	0
Nov. 2020	1.6	10,200	92.0	180	2630	200	5
Feb. 2021	1.5	7,530	94.2	300	2813	200	5
Apr. 2021	1.5	7,960	98.5	–	–	200	5
Sep. 2021	–	6,920	99.1	435	15299	200	5
Nov. 2021	1.3	6,600	101	570	5884	100	5
Jan. 2022	1.4	10,670	97.4	293	1298	100	5

WBC, white blood cell count; MCV, red cell mean corpuscular volume; 6-TGN, red cell thioguanine nucleotide concentration; AZA, azathioprine dosage per day; -, not measured.

## Discussion

In 1996 then 2010, two influential clinical trials with triple therapy of MMF/Pred/CyA showed superior results to AZA**(** 11, 12 **).** At that time, most transplant centers worldwide replaced their immunosuppressive regimen from AZA to MMF. However, a new microemulsion formulation of cyclosporine (Neoral-CyA) appeared, with improved pharmacokinetics.

A large multicenter, prospective, and randomized trial published in 2004 by Remuzzi et al. (13) compared MFF with AZA for acute rejections and adverse events in 168 recipients of cadaver kidney transplants. MMF or AZA treatments were run for six months in combination with Neoral-CyA/Pred (Phase A), and for another 15 months without Pred (Phase B). The primary endpoint was the occurrence of acute rejection episodes. Interestingly, the results showed no significant difference in rejection rates for MMF versus AZA: for Phase A, which included the steroid therapy, the MMF group exhibited a rejection rate of 34%, while the rejection rate for the AZA group was 35% (not significantly different). For Phase B, 89 patients treated without steroids, the performance of AZA was superior: the rejection rate for MMF co-therapy was 16%, while it was 12% for AZA (significantly lower). The authors concluded that MMF had no advantage over AZA to inhibit renal transplant rejection, but MMF is far more expensive. More recently, in 2021, this same group again reported that in a larger and longer-term trial, cadaver donor kidney transplant recipients on low-dose CyA and no steroids experienced no significant benefits of MMF over AZA. They suggested that AZA could safely replace MMF with much lower costs (14).

Shah et al. (15) undertook the first analysis comparing the effects of Pred/Neoral-CyA with either MMF or AZA on the outcomes of paired renal grafts in the United Kingdom. The study included 238 deceased donors from 1999 to 2002 who donated one kidney to a patient treated with MMF and the other kidney to a patient treated with AZA. They found no graft survival differences between MMF and AZA, but higher rejection rates with MMF. A similar superior outcome for AZA over MMF was again reported by Shah et al. (16) in a retrospective study of 10-year renal graft survival.

The calcineurin inhibitor tacrolimus (or FK-506) was licensed for liver transplantation in 1994 and subsequently for other organs, and almost displaced CyA from the market. Schold et al. (17) assessed graft survival among renal transplant patients receiving Pred/tacrolimus with either AZA or MMF and found that AZA co-therapy provided better graft survival and lower rejection rates than MMF. Similarly, McNeil et al. (18) did not observe significant differences in the incidence of acute rejection or bronchiolitis obliterans syndrome in lung transplant recipients treated with MMF or AZA. Beissert et al. (19) demonstrated that AZA and MMF had similar efficacy for the treatment of bullous pemphigoid.

It is worthwhile to cite that other studies have evaluated the benefit of using AZA for the treatment of other conditions, such as ANCA-associated vasculitis, lupus nephritis, and IBD (20–22), but none of them used TPMT or thiopurine metabolite monitoring, which may have produced inaccurate results favoring drug regimens other than AZA for treating those conditions.

The beneficial effect of allopurinol co-therapy with AZA in organ transplantation was first demonstrated in 1993 by Chocair et al. (6). Only one episode of acute rejection was observed among the 12 allopurinol-treated patients, whereas 11 of 12 in a control group had rejection episodes, seven with two or more crises. Several other early studies confirmed that TPMT, combined in some cases with therapeutic drug monitoring, improved the efficacy of AZA in organ transplant. It is important to emphasize that in none of these studies was the dose of AZA adjusted according to blood levels of its metabolites, nor was TPMT assessed.

Subsequently in 1999, Chrzanowska & Krzymanski (23) compared 21 renal transplant patients on AZA triple therapy with six transplanted patients on AZA triple therapy plus allopurinol. The median AZA dosage (mg/d) was considerably lower when allopurinol was included: 33 mg/d AZA (range 25 - 50) versus 68 mg/d (25 – 100) for normal triple therapy. Despite the lower AZA dose, the median 6-TGN was significantly higher for the allopurinol co-therapy group: 363 units (129 - 623) versus 122 units (< 60 - 288) for normal triple therapy (p < 0.005).

AZA/allopurinol co-therapy was extended to inflammatory bowel disease in 2005 by Sparrow et al. (9) They were the first to show for patients resistant to AZA therapy, who tended to ‘shunt’ the drug away from 6-TGN production and towards the TPMT-driven ‘methylation pathway’ (producing 6-MMP), that allopurinol reversed this trend, with red cell 6-TGN levels increasing from a mean of 186 ± 18 to 385 ± 42 pmol/8x10^8^ RBC (p < 0.001), while red cell 6-MMP decreased almost ten-fold. Sparrow et al. concluded, “the addition of allopurinol to thiopurine non-responders with preferential shunting to 6-MMP metabolites appears to be an effective means to shift metabolism” (i.e. towards 6-TGN).

The mechanism of how allopurinol seemingly inhibits the AZA methylation path remained unknown for many years, as allopurinol was known only for its inhibition of uric acid production and prevention of gout. However, this conundrum appears to have been solved, when Blaker et al. demonstrated in 2013 that the mechanism involved TPMT inhibition (24).

Ansari et al. (25) has demonstrated in a series of publications that therapy using allopurinol combined with low-dose AZA is an effective strategy for overcoming thiopurine hepatotoxicity, while providing safe and effective long-term therapy for treating inflammatory bowel disease, particularly when used with TPMT prediction of initial dosage, and provided that myelotoxicity is monitored. Importantly, this group has also demonstrated that allopurinol-AZA therapy is safe during pregnancy, which is an advantage over mycophenylate formulations (26).

AZA is used in treating serious illnesses that often require polytherapy, a fact that complicates the elucidation of the cause of drug-related side effects. Thus, the four patients specially selected for this report were not subjected to multiple drug therapies. Two of them had clinical complications, acute pancreatitis, and severe diarrhea, strongly attributed to toxic levels of 6-TGN (**Figures 1**, **2**), despite using doses considered normal for azathioprine. Monitoring of 6-TGN provided an important method for determining the problem, i.e. high 6-TGN, for these patients. Bergan et al. (5) in 1998 monitored 6-TGN for patients on triple therapy with high dose AZA, and were able to regulate myelotoxicity while reducing acute rejection episodes.

Acute pancreatitis caused by AZA is a relatively common complication in renal transplantation (27, 28), and it also occurs in IBD patients (29, 30). In both types of patients a reduction of the AZA dose in patients with high 6-TGN, regardless of the administered azathioprine dose, may alleviate this adverse event (31). To the best of our knowledge, AZA-related acute pancreatitis was never attributed to 6-TGN levels, as we believe that happened to our patient, and is regarded as an idiosyncratic reaction.

Diarrhea attributed to AZA is a rather rare in renal transplantation. Nonetheless, for one of our cases it appeared to be associated with high 6-TGN, and it resolved after discontinuing AZA. Association of high 6-TGN with gut sensitivity has been reported elsewhere, for IBD patients, and diarrhea has long been an often severe complication for IBD patients receiving AZA (32). However, some IBD cases appear to be intolerant to AZA but not 6-mercaptopurine (31), suggesting that a proportion of gut sensitivity cases may be caused by the imidazole moiety of AZA (33). Thus, withdrawal or reduction of AZA, or swapping to 6-mercaptopurine, may be especially relevant for patients with IBD with diarrhea and other gut sensitivities. An emerging alternative for such cases is the revival of low-dose thioguanine, which produces 6-TGN without the involvement of 6-MMP or imidazole (34). Recent reports described that the use of thioguanine in instead of 6-MP or AZA for patients with a “shunter” phenotype, may be a safe and effective therapy (35, 36). A study from United Kingdom evaluated the use of thioguanine in 193 IBD patients along a median follow-up treatment of 23 months. The 12-month clinical response rate was 65% and elevated liver tests, myelotoxicity and rash were observed in 6%, 7% and 5% of the patients, respectively, emphasizing the safety and efficacy of this therapy (37). This may become a new thiopurine therapy for transplantation.

The third patient presented here had undetectable levels of 6-TGN despite the use of 200 mg/d of azathioprine, possibly contributing to the triggering of acute cell rejection episodes in the first three months after transplantation. For these patients, AZA/allopurinol co-therapy was indicated.

The thiopurine drugs—mercaptopurine, azathioprine and thioguanine—were all invented by one woman, Gertrude Elion. She also invented allopurinol, specifically to improve the efficacy of mercaptopurine in treating leukemia (1). She would have been pleased at the continuing success of these drugs in alleviating human suffering.

## Data Availability Statement

The original contributions presented in the study are included in the article/Supplementary Material. Further inquiries can be directed to the corresponding author.

## Ethics Statement

Ethical review and approval was not required for the study on human participants in accordance with the local legislation and institutional requirements. The patients/participants provided their written informed consent to participate in this study.

## Author Contributions

PC: Conception and design, acquisition of data, analysis and interpretation of data, drafting the article, revising the article critically, acquisition of funding, supervision of the research group. MN: Interpretation of data, drafting the article, revising the article critically. SM: analysis and interpretation of data, revising the article critically. PN: analysis and interpretation of data, drafting the article, revising the article critically. VS: analysis and interpretation of data, drafting the article, revising the article critically. ÉO: analysis and interpretation of data, revising the article critically. LVB: analysis and interpretation of data, revising the article critically. AB: analysis and interpretation of data, revising the article critically. JD: analysis and interpretation of data, drafting the article, revising the article critically. FS: analysis and interpretation of data, revising the article critically. AC-N: analysis and interpretation of data, drafting the article, revising the article critically. All author approved the final version of the manuscript.

## Funding

Hospital Alemão Oswaldo Cruz

## Conflict of Interest

The authors declare that the research was conducted in the absence of any commercial or financial relationships that could be construed as a potential conflict of interest.

## Publisher’s Note

All claims expressed in this article are solely those of the authors and do not necessarily represent those of their affiliated organizations, or those of the publisher, the editors and the reviewers. Any product that may be evaluated in this article, or claim that may be made by its manufacturer, is not guaranteed or endorsed by the publisher.
